# Improved Osseointegration of a TiNbSn Alloy with a Low Young’s Modulus Treated with Anodic Oxidation

**DOI:** 10.1038/s41598-019-50581-7

**Published:** 2019-09-27

**Authors:** Tomonori Kunii, Yu Mori, Hidetatsu Tanaka, Atsushi Kogure, Masayuki Kamimura, Naoko Mori, Shuji Hanada, Naoya Masahashi, Eiji Itoi

**Affiliations:** 1 Department of Orthopaedic Surgery, Tohoku University Graduate School of Medicine, Sendai, Japan; 2 Department of Diagnostic Radiology, Tohoku University Graduate School of Medicine, Sendai, Japan; 3 Institute for Material Research, Tohoku University, Sendai, Japan

**Keywords:** Bone, Biomedical materials

## Abstract

Ti6Al4V alloy orthopedic implants are widely used as Ti6Al4V alloy is a biocompatible material and resistant to corrosion. However, Ti6Al4V alloy has higher Young’s modulus compared with human bone. The difference of elastic modulus between bone and titanium alloy may evoke clinical problems because of stress shielding. To resolve this, we previously developed a TiNbSn alloy offering low Young’s modulus and improved biocompatibility. In the present study, the effects of sulfuric acid anodic oxidation on the osseointegration of TiNbSn alloy were assessed. The apatite formation was evaluated with Scanning electron microscopy, X-ray diffraction, X-ray photoelectron spectroscopy and transmission electron microscopy analyses. The biocompatibility of TiNbSN alloy was evaluated in experimental animal models using pull-out tests and quantitative histological analyses. The results of the surface analyses indicated that sulfuric anodic oxidation induced abundant superficial apatite formation of the TiNbSn alloy disks and rods, with a 5.1-µm-thick oxide layer and submicron-sized pores. *In vivo*, treated rods showed increased mature lamellar bone formation and higher failure loads compared with untreated rods. Overall, our findings indicate that anodic oxidation with sulfuric acid may help to improve the biocompatibility of TiNbSn alloys for osseointegration.

## Introduction

Ti6Al4V alloy is a biocompatible material and resistant to corrosion, and Ti6Al4V alloy orthopedic implants are widely used^1^. However, the elasticity modulus of Ti6Al4V (Young’s modulus: 110Gpa) is appreciably higher compared to cortical bone (10–30 GPa)^2^. In medical practice, differences of the Young’s modulus between femoral implants used in total hip arthroplasty and that of human cortical bone may cause disproportionate stress distribution, leading to pain in the thigh^3,4^. For the resolution of the problem, previous reports described about the development of various β-type titanium alloys with a lower Young’s modulus for use in biomedical devices^5–19^. For example, β-type TiNbSn alloy which has a Young’s modulus <50 GPa can reduce the possibility of stress shielding and thigh pain^12^. However, TiNbSn alloys are unique in that heating to temperatures >673 K (~400 °C) can gradually increase their stiffness and Young’s modulus^20^. This adjustable feature allows titanium alloys to offer greater bonding strength with bone when the Young’s modulus is lowered^9,10,12^. Our previous reports described that TiNbSn alloy showed better biocompatibility and bone induction ability compared to Ti6Al4V alloy in an experimental model^21^. Furthermore, tibial fracture healing in mice and rabbits was enhanced using intramedullary nails made from TiNbSn alloy with a lower Young’s modulus (45 GPa) compared with Ti6Al4V alloy and TiNbSn with a slightly higher Young’s modulus (78 GPa)^22,23^.

Plasma-sprayed coating, hydrothermal treatment with hydrogen peroxide, and alkali-heating treatment has been performed for surface modifications of alloys to improve bone integration^24–26^. Among these, superficial apatite formation of titanium alloys is induced by anodic oxidation (AO)^24,25^. We previously reported that improved apatite formation was shown on the surface of TiNbSn alloy treated by acetic acid AO and following hot water in simulated body fluid (SBF) without changing the low Young’s modulus, and increased bone bonding and higher biocompatibility for osseointegration were indicated^27,28^. While disks can be treated with AO by 2.0 M of acetic acid and electrically controlled at a constant voltage of 200 V and current of 50 mA/cm2, rods require a much higher constant voltage of 500 V and a current of 50 mA/cm^2 ^^27,28^. Therefore, safer and more efficient methods are necessary to improve apatite forming ability and osseointegration on TiNbSn alloy surface.

AO with sulfuric acid requires a lower voltage than AO with acetic acid, and is more efficient^29^. Treatment with a sulfuric acid AO and subsequent annealing at 500 °C to 650 °C for 5 hour in the air produced superficial apatite formation of pure titanium (CP–Ti) in SBF^30–32^. In these studies, subsequent annealing at temperatures >500 °C was required to induce the positive charge on the surface of TiO_2_ and apatite formation after soaking in SBF^31,32^. However, annealing at 500 °C may increase the elasticity modulus of the TiNbSn alloy, as annealing at temperatures <100 °C is required for TiNbSn alloy to maintain a lower Young’s modulus^20^. Therefore, a new method of AO with subsequent annealing at <100 °C and at a lower voltage is required for TiNbSn alloy.

The aim of present study is to examine *in vitro* apatite formation and *in vivo* osseointegration of a TiNbSn alloy treated with AO with sulfuric acid at a lower voltage, followed by annealing at 80 °C or hot water treatment. We hypothesized that AO with sulfuric acid would ameliorate apatite formation and osseointegration in comparison with untreated TiNbSn alloy.

## Results

### Surface analyses

**Figure 1:**
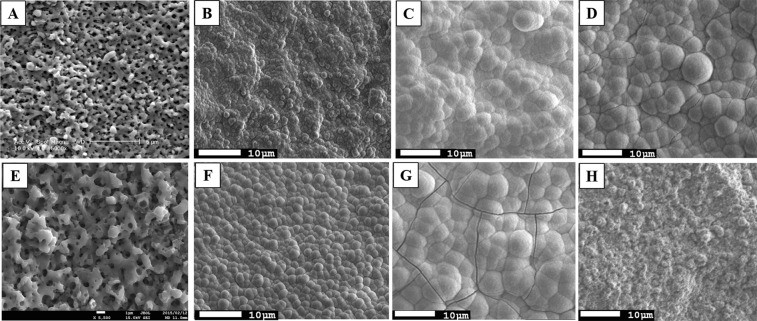
Scanning electron microscopy (SEM) images of apatite formation on anodic oxidation treated CP–Ti and TiNbSn alloy disks. Representative SEM micrograph images of anodic oxidation (AO)-treated pure titanium (CP–Ti; control) disks and TiNbSn alloy disks. (**A**) AO-treated CP–Ti and (**E**) AO-treated TiNbSn. Representative images of SEM micrographs of CP–Ti and TiNbSn disks after AO treatment, AO + annealing treatment, and AO + hot water treatment, followed by 7-day incubation in HBSS. (**B**) AO-treated CP–Ti; (**F**) AO-treated TiNbSn; (**C**) AO + annealed CP–Ti; (**G**) AO + annealed TiNbSn; (**D**) AO + hot water-treated CP–Ti; and (**H**) AO + hot water-treated TiNbSn.

**Figure 2:**
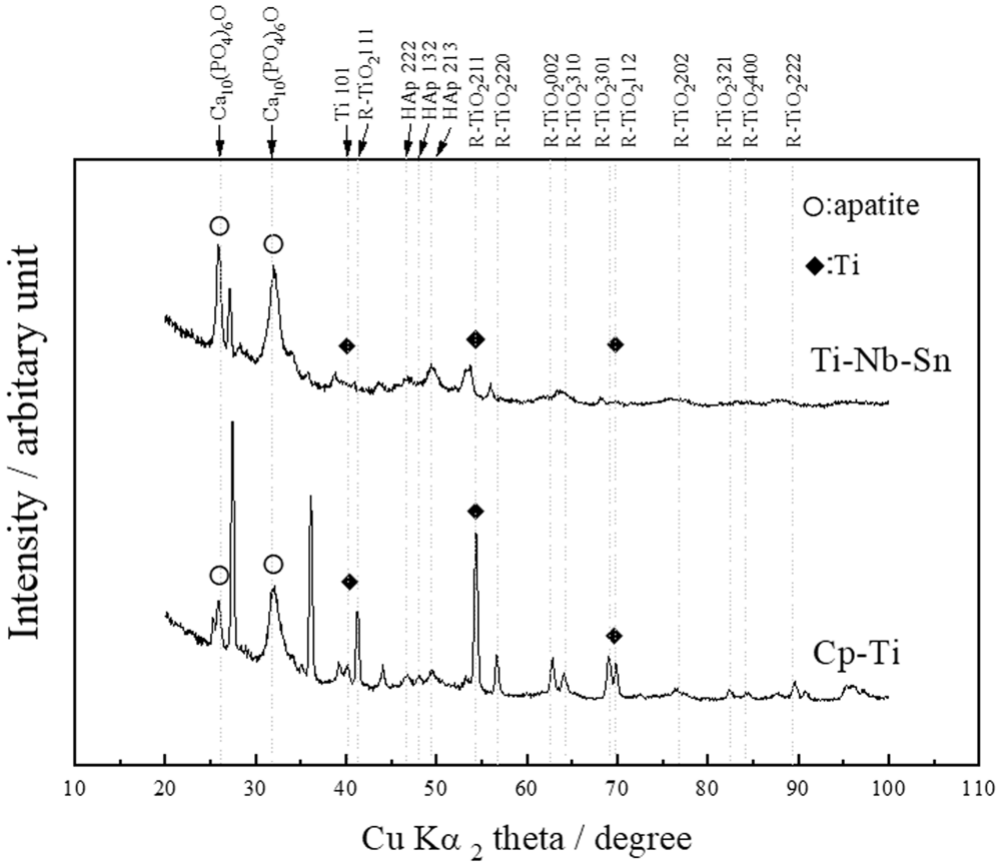
X-ray diffraction (XRD) analyses of apatite formation. XRD analyses of anodic oxidation (AO)-treated pure titanium (CP–Ti; control) and TiNbSn disks subsequent 7-day incubation in HBSS. Crystalline apatite formation were observed in both AO-treated CP–Ti and TiNbSn alloy disks.

**Figure 3:**
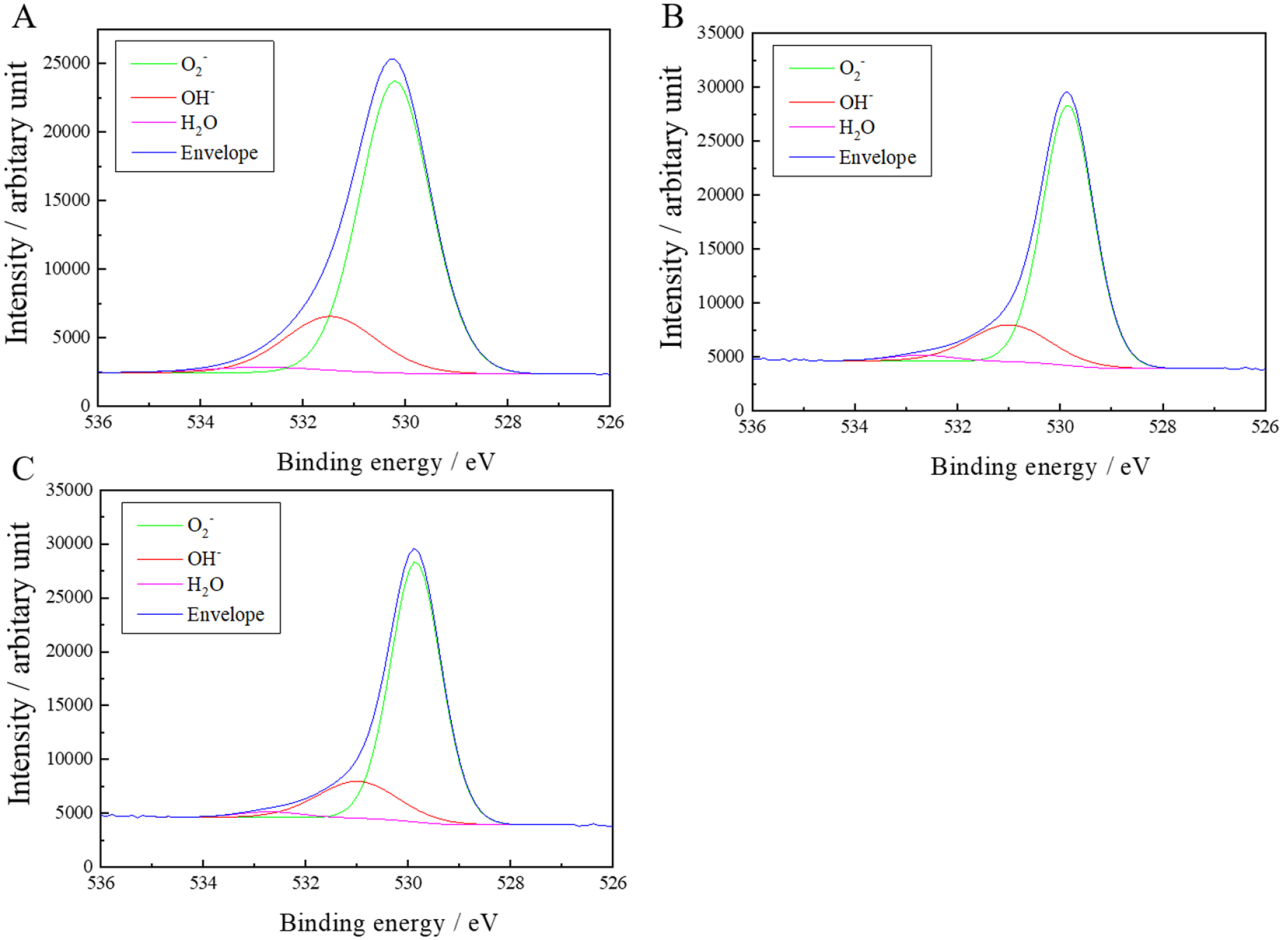
X-ray photoelectron spectroscopy (XPS) analysis of the surfaces of the TiNbSn alloys. O 1 s XPS profiles of treated TiNbSn disks. (**A**) Anodic oxidation (AO)-treated TiNbSn disk; (**B**) AO + hot water-treated TiNbSn disk; and (**C**) AO + annealed TiNbSn disk. No apparent differences were observed in the fraction of hydroxyl groups of the treated surfaces.

TiNbSn alloy and CP–Ti (control) disks with dimensions of 10 mm in diameter and 2 mm in thickness were polished using SiC grading paper followed by washing with an ultrasonic cleaner in ethanol. Figure 1 shows plan-view SEM images of AO-treated (A, B, E, F), AO + annealed (C, G), and AO + hot water-treated (D, H) on Ti (A–D) or TiNbSn (E–H) substrate before (A, E) and after (B, C, D, F, G, H) soaking in Hank’s Balanced Salt Solution (HBSS) for 7 days. The ionic concentrations of HBSS were comparable to human blood plasma as well as available SBF. A porous microstructure with 1–2 μm pores in diameter was observed in the as-anodized oxide regardless of the substrates (A, E). Granular-shaped microstructures are observed in the AO-treated (B, F), AO + annealed (C, G), and AO + hot water-treated (D, H) irrespective of substrate and subsequent treatment. Figure 2 shows the X-ray diffraction (XRD) profiles of the anodic oxide on CP–Ti and TiNbSn alloy, these results reveal that all detected peaks are assigned to rutile-structured TiO_2_, bcc-Ti and hydroxyl-apatite (Ca_10_(PO_4_)_6_O). The survey X-ray photoelectron spectroscopy (XPS) spectra of the anodic oxides showed peaks due to Ti, Nb, Sn, O, S and C, and a weak N peak. Peaks corresponding to C and N are originated from contamination during sample preparation and air exposure. The anodic oxide on TiNbSn alloy was composed of TiO_2_, Nb_2_O_5_, and SnO or SnO_2_, as described elsewhere^33^. Figure 3 exhibits O 1 s XPS spectra of as-anodized oxide (A), annealed anodic oxide (B), and hot water-treated anodic oxide (C). These spectra show asymmetrical shape with a shoulder peak extending toward higher binding energies, which was attributable to the hydroxyl groups. The O 1 s XPS spectrum has distinct three peaks of metal oxide, hydroxyl groups, and water molecules. Semi-quantitative analysis using Casa XPS software (www.casaxps.com) concluded that the atomic fraction of metal oxide, hydroxyl groups and water molecules on the surface of the oxide were 58.1/11.0/0.10, 57.2/11.9/0.10 and 60.2/8.7/0.5 for as-anodized, annealed and hot-water treated oxide, respectively. Hot water treatment increases the fraction of metal oxide and water molecules, while no change was found in annealing treatment.

### Pull-out tests for untreated and AO-treated TiNbSn rods

**Figure 4:**
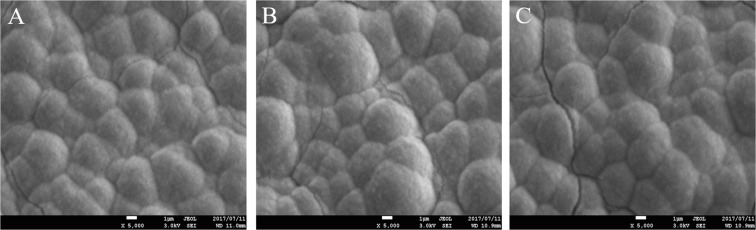
Scanning electron microscopy (SEM) images of the superficial apatite formation of TiNbSn alloy rods. Representative SEM micrograph images of anodic oxidation (AO)-treated (**A**), AO + annealed (**B**), AO + hot water-treated rods (**C**) subsequent 7-day incubation in HBSS. All groups showed abundant apatite formation.

Figure 4 shows representative SEM images of AO-treated, AO + annealed, and AO + hot water-treated rods following 7-day incubation in HBSS. All three treatments led to abundant apatite formation.

**Figure 5:**
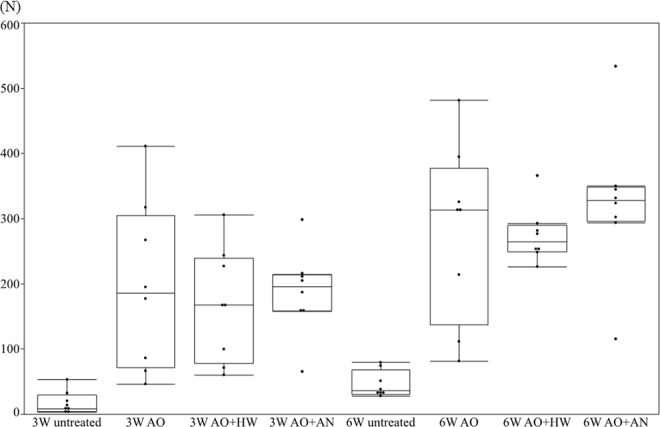
Assessment of bone bonding strength with pull-out tests. At 3 and 6 weeks after rod implantation, the failure loads of treated and untreated TiNbSn rods were shown. The results of pull-out tests of AO-treated, AO + annealed and AO + hot water-treated rods showed higher failure loads compared with the untreated rods both at 3 and 6 weeks. Results are expressed as the median and interquartile range (n = 8 rabbits per group).

In the *in vivo* analysis, we tested the failure load of the rods using pull-out tests at 3 and 6 weeks after implantation. At 3 weeks after rods insertion, the failure loads of the AO-treated, AO + annealed, and AO + hot water-treated rods were 186.1 (233.3) N, 167.4 (161) N, and 195.9 (55.5) N, respectively, which were all higher than that of untreated rods [8.31 (25.3) N]. A similar pattern was seen at 6 weeks in the failure loads for untreated rods compared with treated rods [untreated rods, 35.9 (38.3) N vs. AO-treated, 313.5 (240.3) N; AO + annealed, 264.7 (40.7) N; and AO + hot water-treated 327.6 (52.9) N (Fig. 5)]. These results indicated that AO treatment with sulfuric acid induced better apatite formation and osseointegration of TiNbSn alloy rods. However, annealing treatment and hot water treatment after AO also showed no synergistic effects on formed bone.

### Histological evaluation

Considering the results of the pull-out tests and surface characterization, we concluded that little differences existed between the AO-treated, AO + hot water-treated, and AO + annealed groups; therefore, we only compared the AO-treated and untreated TiNbSn groups in the histological analyses.

**Figure 6:**
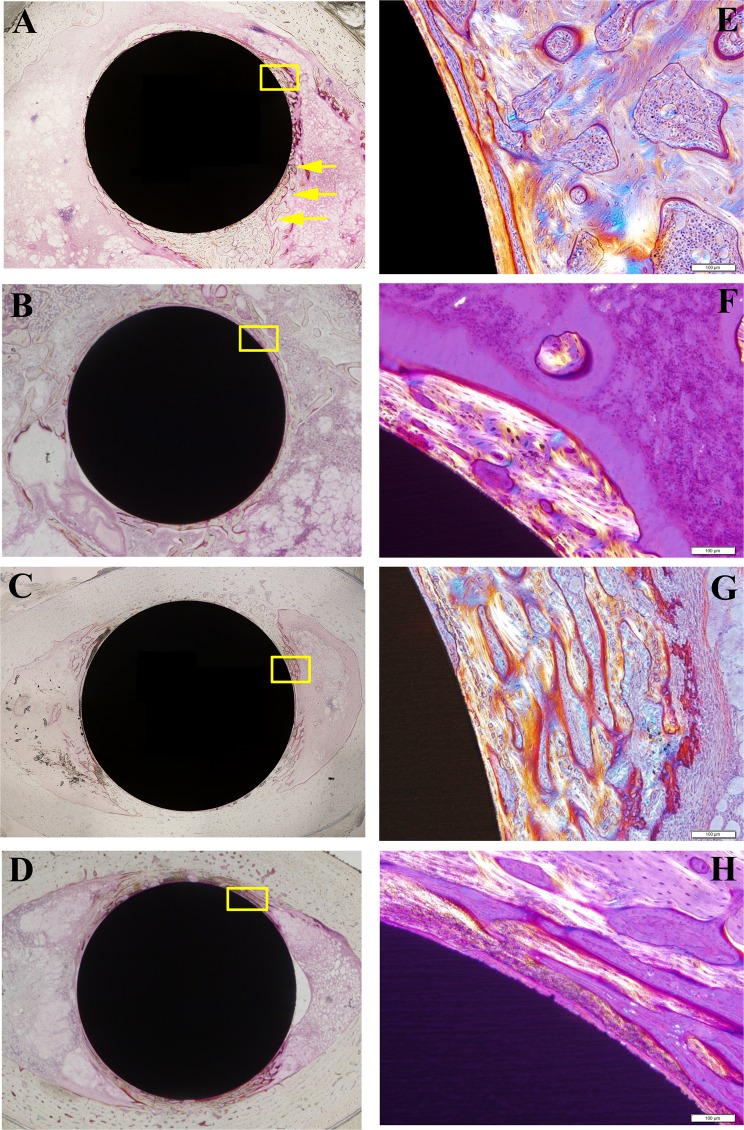
Histological images of new bone formation around the TiNbSn rods. Representative histological images of untreated and AO-treated TiNbSn alloy rods implanted in rabbit femurs. (A–D) panels show lower magnification images. (E–H) panels show higher magnification images visualized using a polarization microscope of the rectangular areas indicated in (A–D) panels. Mature lamellar bone (arrow heads) was observed in the AO-treated TiNbSn group. (**A**,**E**) Images of distal femur of an AO-treated rod; (**B**,**F**) Images of distal femur of an untreated rod; (**C**,**G**) Images of proximal femur of an AO-treated rod; and (**D**,**H**) Images of proximal femur of an untreated rod.

**Figure 7:**
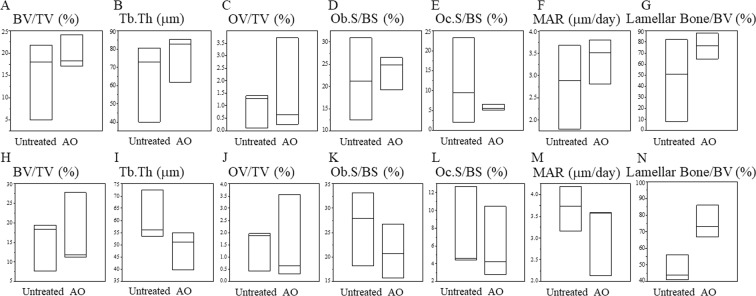
Quantitative histomorphometric analyses of new bone formation. (A–G) panels indicated the results of distal femur and (H–N) panels indicated the results of proximal femur. The lamellar bone formation in the proximal area is higher in the AO-treated TiNbSn alloy rods compared with that of the untreated rods. Results are expressed as the median and interquartile range (n = 3 rabbits per group). BV/TV, bone volume/tissue volume; Tb.Th, trabecular thickness; OV/TV, osteoid volume/tissue volume; Ob.S/BS, osteoblast surface/bone surface; Oc.S/BS; osteoclast surface/bone surface; MAR, mineral apposition rate.

Figure 6 shows representative histological sections for each group. No differences were observed in the volume of new bone formation between the groups. However, AO-treated group showed greater lamellar bone formation in the proximal area of femur, in comparison with that of the untreated group. The results of quantitative histological analyses are shown in Fig. 7. In the proximal area of femur, the ratio of lamellar bone to total bone was higher in AO-treated [73.2% (19.2%)] compared with untreated [44.0% (15.2%)] rods. At the distal end, there was a tendency toward better lamellar bone formation in the AO-treated group. No differences were shown in the other parameters of histomorphometric analysis between the two groups. These results indicated that AO treatment with sulfuric acid improved the formation of mature lamellar bone on the surface of AO-treated TiNbSn rods.

### Transmission electron microscopy (TEM)

**Figure 8:**
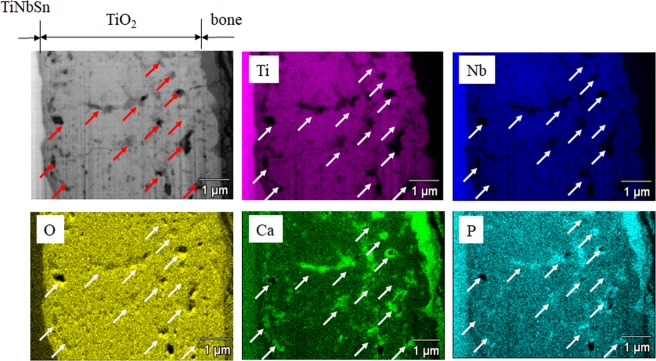
Transmission electron microscopy (TEM) image of titanium oxide layer on the boundary surface of the rod and bone in AO-treated TiNbSn alloy. TEM image of titanium oxide layer on the boundary surface of the rod and bone, and mapping of elements of Ti, Nb, O, Ti, Ca, and P in the same region. Submicron-sized pores were observed and are indicated by arrows. Mapping revealed that both Ca and P segregated and inserted into the pores.

Cross-sectional High-angle Annular Dark-field Scanning Transmission Electron Microscopy (HAADF-STEM) observation and energy-dispersive X-ray spectroscopy analysis were conducted for the specimens taken from the interface vicinity between the bones and implanted anodized TiNbSn rod at 6 weeks after implantation. Figure 8 shows the HAADF-STEM image and elemental mapping of Ti, Nb, O, Ca, and P of the oxide layer. Abundant submicron-sized pores (marked by arrows) were observed over the thickness of the oxide layer. It revealed incorporation of bone ingredients of Ca and P in TiO_2_ layer and segregation in the pores. Here, it is noted that the presence of P in the TiNbSn was attributed to the close characteristic X-ray energies of Nb Lα (2.166 keV) and P Kα (2.013 keV).

## Discussion

We previously reported that TiNbSn alloy with by acetic acid AO treatment and subsequent hot water treatment induced hydroxyapatite formation in HBSS and increased bone bonding strength in rabbit femurs^27^. We also reported that bone components, Ca and P, penetrated titanium oxide layer of TiNbSn alloy treated with acetic acid AO plus hot water, with photo-induced properties^28^. Furthermore, the thickness and crystallization quality of the anodic oxide produced by AO with acetic acid treatment could be increased by hot water treatment or annealing^28^. In this study, we showed that AO treatment with sulfuric acid, without high-temperature annealing or hot water treatment, produced apatite formation in HBSS and improved bone integration in rabbit femurs. Furthermore, compared with AO with acetic acid, AO with sulfuric acid led to increased anodic oxide thickness and better crystallization of the anatase form of TiO_2_, and was unaffected by hot water treatment or annealing. Finally, TEM was used to further examine the boundary surface of the bone and TiNbSn alloy. Around the pores of TiO_2,_ there was abundant deposition of calcium and phosphorus. The pore size of TiNbSn treated with AO with sulfuric acid was larger than observed following treatment with acetic acid. This larger pore size allowed for better deposition of Ca and P; therefore, we postulated that prostheses with thicker anodic oxide and larger pores may provide an optimal environment for improved apatite formation, allowing for stronger binding with human bone.

The results of implant extraction test indicated that the failure loads of AO-treated, AO + annealed, and AO + hot water-treated TiNbSn alloy rods were greater than that of untreated TiNbSn alloy. However, histological analysis revealed that the volume of new woven bone formed around the rod was similar for the AO-treated and untreated TiNbSn alloy. Analysis using a polarization microscope showed that treated rods had higher proportions of lamellar bone, which is the mature bone that provides stronger bonding than immature woven bone, compared with untreated rods. These findings corresponded with the results of extraction tests.

Intriguingly, pull-out analysis showed no apparent differences among AO-treated, AO + hot water-treated, and AO + annealed rods. We propose that AO with sulfuric acid stimulates apatite integration around implanted TiNbSn alloy, irrespective of annealing and hot water treatment. We conclude that bone remodeling may be enhanced by better biocompatibility of TiNbSn alloy treated with sulfuric acid AO compared with untreated alloy, as indicated by the greater mature lamellar bone formation in the treated samples.

The higher bonding strength in AO-treated, AO + annealed, and AO + hot water-treated rods compared with untreated rods at 3 and 6 weeks after rod embedding indicated strong cohesion of bone and implanted TiNbSn alloy (Fig. 5), and could be attributable to anodic oxide on the surface of TiNbSn alloy rod. Osseointegration of titanium oxide has been reported extensively^34,35^, whereas the role of oxide has not yet been delineated. We propose that the superior osseointegration of TiO_2_ prepared by anodization may be attributed to highly crystallized TiO_2_ with pore sizes that facilitate the incorporation of elements crucial to bone formation (Ca, P) beyond the limit of solubility. Previously reported pseudo-ternary equilibrium phase diagrams of TiO_2_ and CaO^36^ or P_2_O_5_^37^ indicate that both calcium and phosphorus are in equilibrium with titanium oxide. However, submicron-sized pores within the TiO_2_ layer (likely originating from oxygen that evolves in the chemical reaction) also act as “sink sites” for these elements. Strong acids such as sulfuric acid generate large quantities of protons due to their high ionization constant (1.99)^38^, thereby promoting AO. Superoxide (O^2−^) generated during AO reacts with Ti^4+^ to form TiO_2_, whereas excessive O^2−^ forms pores within the oxide layer. This is in contrast to the anodic oxide prepared using an acetic acid electrolyte^28^, in which anodization progression is “sluggish,” and the frequency of pore appearance in the oxide layer is low due to a lower electrolytic dissociation constant of 4.76^38^. Furthermore, high sulfuric acid concentration in the electrolyte promotes crystallization of TiO_2_ due to self-heating during anodization, as indicated by the formation of a high-temperature, stable rutile-structured TiO_2,_ and a substantial rise in temperature of the sulfuric acid electrolyte. Highly crystallized TiO_2_ and the abundance of micro-sized pores prepared via anodization in the sulfuric acid electrolyte are beneficial for osseointegration, and eliminate the requirement for subsequent treatments such as annealing or hot-water treatment.

While the elastic anisotropy of β-type titanium alloy reported in the literature, no data of the elastic moduli of α”-type titanium alloys is currently reported. We studied the Young’s modulus of TiNbSn alloy, focusing on the constituent phase and crystal orientation, and concluded that a low elastic modulus originated from β with α” accompanied by evolution of crystal orientation of β 〈110〉 and α” 〈010〉^39^. With thermomechanical treatment using extrusion, rolling, and swaging, this microstructure was invented to Ti–33.6Nb–4Sn alloy (mass percent) which has near β composition with suppressing ω precipitation. Heavy deformation with 91% reduction in rolling induces a precipitation of martensite α”, accompanied by a fiber texture constituted of α” 〈010〉 and β 〈110〉. Subsequent swaging stabilizes α” and evolves the fiber texture, lowering the modulus of elasticity to 40 GPa. Our previous report showed a strain stress curve of the present alloy^40^. The present study revealed a higher tensile strength of heavy deformed alloy composed of β and α”-type Ti compared with that of hot-forged alloy of monolithic β; therefore, we concluded that this was due to stabilization of α” in the β matrix, accompanied by texture evolution.

In conclusion, the present study demonstrated that treatment with AO with sulfuric acid promoted apatite formation and osseointegration in TiNbSn alloy compared with untreated alloy. Furthermore, sulfuric acid treatment avoided the requirement for further treatment of the alloy with hot water or annealing. These results may help to improve the biocompatibility of TiNbSn alloys and improve osseointegration.

## Methods

### Preparation of TiNbSn alloys

The preparation of TiNbSn alloy was described in a previous report^27^. We fabricated Ti-25.4 Nb-9.9 Sn (mass %) alloy ingots by a high frequency induction melting in an Ar atmosphere and re-melting using a vacuum arc melting. Forging ingot was performed at 1100 °C, with subsequent cold rolling and swaging to obtain a rod with a 15-mm diameter. TiNbSn alloy exhibits a Young’s modulus lower than 55 GPa. TiNbSn alloy and grade-2 CP–Ti (control) disks with dimensions of 10 mm in diameter and 2 mm in thickness were polished using SiC grading paper followed by washing in ethanol using an ultrasonic cleaner and dried in an atmosphere, which were provided for the examination of apatite forming in SBF (*in vitro* test). Cylindrical rods with dimensions of 4.5 mm in diameter and 32 mm in length for the pull-out test were prepared from 8-mm diameter rods. The Young’s modulus of the fabricated rod was 45.6 GPa. A protruding portion (4.0 mm in diameter, 6 mm in length) with a transverse hole for mechanical testing was made at one end, and a capped tapering portion was also made on the other (3 mm end diameter, 1 mm length) in the rods. The tapered end allowed for smooth insertion of the rod into the medullary canal.

### Surface modification

Anodic oxidation was performed for TiNbSn alloy and CP–Ti disks for 30 minutes with 1.0 M sulfuric acid using a constant voltage of 210 V and a current of 50 mA/cm2 at room temperature. These modified disks were designated as “AO-treated disks.” Anodic oxidation was also performed for TiNbSn alloy rods with sulfuric acid using the same method. After AO treatment, disks and rods were subjected to either annealing treatment at 80 °C or hot water treatment, and incubated in 25 mL of HBSS (GIBCO, Grand Island, NY, USA) at 37 °C for 14 days as previously described^27^. After 14 days, the disks and rods were softly rinsed using pure water and desiccated in an incubator for 24 hours.

### Surface analyses

We performed surface analyses of the samples as previously described^27,28^. The observation of the microstructure of the samples incubated in HBSS was performed by field-emission SEM (FESEM; Scanning Electron Microscope SU 8000, Hitachi, Japan) and analyses by XRD (X’Pert diffractometer, PANalytical, Netherlands) with a thin-film geometry arrangement using a 0.5 ° glancing angle and a rotating detector was also performed. The topmost surface of the samples was analyzed by X-ray photoelectron spectroscopy (XPS) equipped with an electron spectrometer (Shimadzu, Kratos AXIS-Ultra DLD, Japan) with monochromated Al Kα radiation at a base pressure of 3.0 × 10^−7^ Pa. The full width at half maximum intensity of the Ag 3d_5/2_ peak was 0.73 eV, and the base pressure of the spectrometer was 6.5 × 10^−8^ Pa. We prepared cross-sectional specimens of the anodic oxides using FIB with an operating voltage of 30 kV (Versa 3d Dual Beam, FEI, USA). The specimens were observed by FE-SEM (SU8000, Hitachi, Japan) with an operating voltage of 5 kV, and TEM (JEM-ARM200F, JEOL, Japan) with an operating voltage of 200 kV.

### Animal experiments

A rabbit femur titanium alloy-implanted model previously reported was refencened^27^. Male adult Japanese white rabbits were obtained from Charles River Laboratories Japan, Inc. The animal were housed at the Animal Unit of Tohoku University Medical School and individually kept in cages (60 cm width × 51 cm depth × 35 cm height) with specific pathogen free environment. The Animal Research Committee of Tohoku University reviewed and approved all animal protocols, and we performed all animal experiments following the relevant guidelines and regulations. Rabbits with 3.0–3.5 kg of body weight were used in the experiments. After intramuscular injection of ketamine (25 mg/kg), intravenous injection of ketamine (10 mg/kg) and xylazine (3 mg/kg) were performed as anesthesia. Intravenous injection of cefazolin (30 mg/kg) was given prior to surgery. AO-treated, AO + annealed, AO + hot water-treated, and untreated TiNbSn alloy rods were implanted into the bone marrow cavity of rabbit femurs. Postoperative wound conditions (healing, redness, and soft tissue edema) were assessed visually. Animals were sacrificed with pentobarbital (120 mg/kg) via intravenous administration at 3 and 6 weeks after rod implantation.

### Pull-out test for bone bonding strength

Pull-out tests were performed at 3 and 6 weeks after implantation, as previously described^27^. Briefly, the distal end of the femur was cut to expose the transverse hole in protrusion for connecting to the testing machine (Autograph, Shimadzu, Japan). We constructed a connecting device for the testing machine to pull the pull the rods with transverse hole vertically. The measurement of failure load was performed for each of group (AO-treated, AO + annealed, AO + hot water-treated, and untreated rods) (n = 8 for each group).

### Histomorphometric analysis

We performed histomorphometric analysis at 6 weeks after implantation of the rods into the rabbits, as previously described^27^ (n = 3 for each group). Briefly, at 7 and 2 days prior to sacrifice, the femurs of rabbits were double-labeled with 20 mg/kg tetracycline (Sigma, Germany) and 10 mg/kg calcein (Dojindo Laboratories, Japan) via subcutaneous injection, respectively. Excision of the femurs with implants were performed, and samples were immediately soaked in 70% ethanol for 5 days. Femurs were soaked with Villanueva bone stain reagent for 6 days. Dehydration in ascending grades of ethanol and defatting in an acetone/methyl methacrylate monomer solution (1:3) was performed for the samples. After these procedures, the samples were embedded in methyl methacrylate (Wako Chemicals, Japan) and cut vertically to the long axis of the implant at proximal and distal levels of the femur, using a precision bone saw. Cross-sections (200 μm) were obtained without decalcification. Mounted sections on plastic slides were ground with a precision lapping machine (Maruto, Japan). The thickness of ground specimens was 40 μm and monitored using optical and polarising microscopes. We performed histomorphometric measurements with a semiautomatic image analyzing system (System Supply, Ina, Japan) using a method previously described by Frost^41^. The cancellous bone around the implant was estimated, and the following histomorphometric bone parameters were assessed: BV/TV (%), Tb.Th (mm), OV/TV (%), Oc.S/BS (%), Ob.S/BS (%), and MAR (mm/day)^42^. The ratio of lamellar bone to total bone was also measured using a polarization microscope to assess the maturation of bone remodeling in the callus.

### Statistical analysis

Descriptive analyses were performed to assess the pull-out test and histomorphometric analysis, and the median and distribution data of the interquartile range were expressed.
